# Females do not have more injury road accidents on Friday the 13th

**DOI:** 10.1186/1471-2458-4-54

**Published:** 2004-11-16

**Authors:** Igor Radun, Heikki Summala

**Affiliations:** 1 Traffic Research Unit, Department of Psychology, University of Helsinki, PO Box 9, 00014 Helsinki, Finland

## Abstract

**Background:**

This study reinvestigated the recent finding that females – but not males – die in traffic accidents on Friday the 13th more often than on other Fridays (Näyhä S: Traffic deaths and superstition on Friday the 13th. Am J Psychiatry 2002, 159: 2110–2111). The current study used matched setting and injury accident data base that is more numerous than fatality data. If such an effect would be caused by impaired psychic and psychomotor functioning due to more frequent anxiety among women, it should also appear in injury crashes.

**Methods:**

We used the national Finnish road accident database for 1989–2002. To control seasonal variation, 21 Fridays the 13th were compared in a matched design to previous and following Fridays, excluding all holidays, on number of accidents, male/female responsibility for accidents, and the number of dead, injured and overall number of active participants (drivers, pedestrians and bicyclists) as a consequence of the accident.

**Results:**

There were no significant differences in any examined aspect of road injury accidents among the three Fridays, either in females or males. Women were not overrepresented in crashes that occurred on Fridays 13th.

**Conclusion:**

There is no consistent evidence for females having more road traffic crashes on Fridays the 13th, based on deaths or road accident statistics. However, this does not imply a non-existent effect of superstition related anxiety on accident risk as no exposure-to-risk data are available. People who are anxious of "Black Friday" may stay home, or at least avoid driving a car.

## Background

One widely spread superstition is that Friday the 13th brings bad luck. However, the few studies published on human behaviour and its consequences on that day show inconsistent results, whether they be on economic behaviour [1-3] or health risks [4-6]. A recent nationwide study by Näyhä [7] on the 1971–97 death statistics in Finland found that men's deaths did not increase on Friday the 13th but females' did by a factor of 1.61, and by 1.63 when adjusted for age, time period, temperature, and extra Poisson variation. The author's conclusion was that Friday the 13th may be a dangerous day for some women, presumably because of anxiety from superstition and, possibly, anxiolytic medications.

This interpretation is not without problems. First, although the author repeatedly refers to driving, it should be noted that he also included water and air traffic accidents. Secondly, as the author pointed out himself, his data included passengers killed in accidents, who typically have no control on the task. Impaired psychic and psychomotor functioning due to anxiety, which could indeed be more frequent in females due to their higher neuroticism rate [8], superstition [9,10] and smaller amount of driving experience [11] should primarily affect safety in cases where females were active traffic participants. Third, weather conditions were controlled by the mean daily temperature obtained from one place close to the population centered midpoint of the country. However, Finland is more than 1000 km in length, located between the 60th and 70th deg of Northern latitude with much variation in weather. The vicinity of the sea increases variation in weather and road conditions even more in the southern coast where the population and traffic are heavily concentrated. Any adjustment based on one location cannot be effective. Fourth, by excluding only Good Fridays the author had a sample of Fridays the 13th without holidays because no major holiday in Finland falls on the 13th of month. However, there are plenty of holidays among all other Fridays with quite different travel patterns and life style. For example, Midsummer Eve always falls on Friday in the second part of June, which gives 27 such days in study period 1971–1997. The Midsummer Eve is a marked peak in alcohol consumption in Finland [12], as well as of crashes of male drivers. Friday can also fall on Christmas day, New Years day, First of May and some other holidays with much reduced traffic volumes and exposure to risk. Finally, in spite of the long study period, the data only included 41 female deaths on 43 Fridays the 13th, which means 16 deaths more than expected from all other Fridays during the study period.

In spite of Näyhä's fairly conservative conclusion, his results have been widely publicised as evidence that superstitious female drivers die on Fridays the 13th [13] in marked contrast to men. Due to the shortcomings listed above, and fairly small sample size, the results deserve reinvestigation to avoid premature conclusions and improper interpretations which tend to promote sexist attitudes about women drivers.

We reinvestigated the case using the national Finnish road accident data base of injury accidents [14] for 1989–2002, all years available in a comparable format. These data also include road-traffic fatalities, and for that part they overlap with Näyhä's study period and data. A matched design was selected which makes it possible to control seasonal effects and to avoid the problems due to holidays. Injury accidents are much more numerous than fatalities. If women's assumed more frequent superstitious (and traffic-related) anxiety indeed would result in attentional and psychomotor dysfunctioning on Fridays the 13th, claimed by Näyhä [7] on the basis of fatality statistics, the effect should also be found in injury crashes.

## Methods

There were 24 Fridays the 13th during the study period. However, three of them were excluded because two were Good Fridays and one followed a Thursday holiday. To control seasonal variation in traffic and weather-type, the remaining 21 Fridays the 13th were compared with the previous Fridays the 6th and the following Fridays the 20th on the number of accidents, male/female responsibility for accidents (police officer judgment), the number of dead, injured and overall number of active participants as a consequence of accident, separately for women and men. Active participants included drivers, bicyclists and pedestrians who actively controlled their motion in traffic and may get involved in crashes. Motor vehicle passengers were excluded. Nine holidays or otherwise unusual control Fridays were replaced by the mean values of the accident variables (e.g. number of accidents, number of injured) from the previous and following years' closest Fridays. For example, Friday the 20th in June 1997 fell on Midsummer holiday eve, and was replaced by mean values gathered from Fridays June 14th, 1996 and June 12th, 1998. This was done to preserve size of the sample. To avoid violating parametric assumptions, the Friedman analysis of variance by ranks [15] was used to test differences across 21 matched triplets of Fridays.

## Results

**Figure 1:**
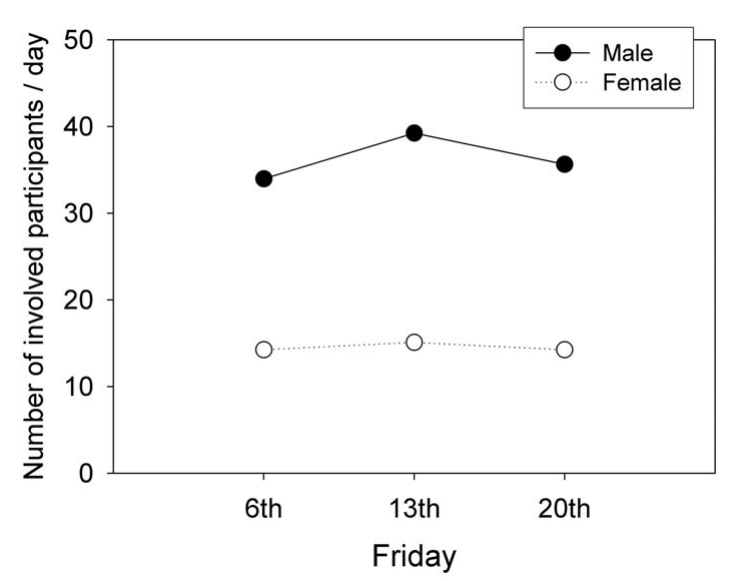
The average daily number of active participants involved in injury road crashes by gender on the Fridays 13th and the preceding and following Fridays for 1989–2002.

Tables 1, 2, 3 present accidents and active participants and victims by gender for the Fridays the 13th and the preceding and next Fridays. Figure 1 depicts daily means of active participants by gender on each of three Fridays.

**Table 1:** The number of injury accidents on Fridays the 13th, the previous (the 6th) and following (the 20th) Fridays. N for the matched triplets of Fridays = 21.

Friday	Total	Daily Mean
6th	542.5*	25.83
13th	608	28.95
20th	546.5	26.02

* Decimal numbers in Tables 1-3 are due to replacement of seven holidays or otherwise unusual control Fridays with the mean values of the variables from the previous and following years' closest Fridays.

**Table 2:** The number of active participants* by gender on Fridays the 13th, the previous (the 6th) and following (the 20th) Fridays.

Female				Male		
Friday	Total	Drivers	Pedestrians and Bicyclists	Total	Drivers	Pedestrians and Bicyclists

6th	299.5	193.5	106	713.5	618	95.5
13th	317	198	119	824	705	119
20th	299	183	116	748.5	661.5	87

* Active participants included drivers, bicyclists and pedestrians who actively control their motion in traffic and may get involved in crashes.

**Table 3:** The number of victims* by gender on Fridays the 13th, the previous (the 6th) and following (the 20th) Fridays.

Female			Male	
Friday	Dead	Injured	Dead	Injured

6th	6 (9)*	196 (290.5)	27.5 (33.5)	308.5 (372)
13th	15 (21)	214 (304)	30 (35)	340 (430)
20th	11 (13)	195.5 (296)	22.5 (25)	329.5 (418.5)

* Numbers for victims refer to active participants while those in parenthesis also include passengers.

Comparisons of 21 triplets showed no significant difference in injury accidents (Friedman χ^2 ^= 3.534, df = 2, p = 0.171); in active participants, for females (χ^2 ^= 0.025, df = 2, p = 0.987) or males (χ^2 ^= 0.173, df = 2, p = 0.917); in injured active participants, for females (χ^2 ^= 1.162, df = 2, p = 0.559) or males (χ^2 ^= 0.532, df = 2, p = 0.767); and in dead active participants, for females (χ^2 ^= 2.735, df = 2, p = 0.255) or males (χ^2 ^= 0.448, df = 2, p = 0.799) among three Fridays. To test the Gender × Day interaction, we also computed female/male ratios of active participants for each Friday of each triplet, and applied the Friedman test to check whether this ratio is systematically higher on Fridays 13th, as expected from Näyhä's [7] results. The ratio was quite similar on each Friday (6th: 0.420, 13th: 0.385, 20th: 0.452; χ^2 ^= 0.400, df = 2, p = 0.819) indicating that, with respect to men, women are not overrepresented in crashes that occur on Fridays 13th. The odds for women being involved in an injury tend to be even somewhat smaller on Friday 13th. There was no such overrepresentation in injured (χ^2 ^= 1.615, df = 2, p = 0.446) or dead (χ^2 ^= 2.1, df = 2, p = 0.350) women among active participants.

Finally, we similarly checked a possible Gender × Day effect in legally responsible crashes (responsibility drawn from police reports), computing female/male ratios of guilty participants for each day and triplet, but did not found any effect(Friedman test, χ^2 ^= 0.514, df = 2, p = 0.774).

## Discussion

This study could not find any indication of overrepresentation of women in injury crashes on Friday the 13th. This is inconsistent with Näyhä's [7] results and conclusions that were based on less numerous deaths statistics (41 women died in all traffic accidents on Fridays 13th 1971–97) compared to injury road traffic accidents (317 active female participants on Fridays 13th 1989–2002), and also inconsistent with earlier British results [4]. Given that women's more frequent superstition and related anxiety would cause unsafe traffic behaviour, injury accidents should increase on Friday the 13th as well as fatalities. This was definitely not the case in the Finnish road accident statistics. Although injury accidents are not reported as completely as fatalities, we do not see any reason for biased reporting on Fridays the 13th. Our analysis did not even show any significant gender effect in fatalities.

It is to be noted that both Näyhä's study [7] and this study are based on aggregated data (number of accidents per day in the country). In contrast to individual level analysis, such data mixes individual confounders and outcomes and, therefore, confounding factors cannot be fully controlled [16]. Our matched countrywide setting is a quasi-experimental design well suited to simple comparisons of crash rates in gender populations which keep constant in each triplet of successive Fridays (see also [4]). We also assume that this design is quite powerful in controlling seasonal variation (e.g. in traffic and weather).

However, our data only implies that, in comparison to men, women are not overrepresented in injury road accidents on Fridays 13th in Finland for 1989–2002. We do not and we cannot conclude anything about women's performance in traffic on Fridays 13th, or about their accident risk (given certain exposure to risk), or about the effect of superstition on those risks. For such conclusions, disaggregated individual level data is needed with detailed information of exposure to risk and respective accident outcome. People themselves adjust their exposure to risk at several levels, while making trip decisions, choosing transport mode, or selecting routes to the destination (see the "multiple sieve model" of accident output [17]). Therefore, those who are really anxious about Friday 13th may stay at home, use public transportation instead of car, avoid rush hours, choose safer routes, or avoid dangerous junctions. But for one left turn while driving, or for one crossing of street while walking, their risk may be higher.

## Conclusion

We conclude that, in the Finnish traffic accident statistics for 1989–2002, females have not incurred more injury (or fatal) road traffic accidents on Fridays the 13th than expected, as a driver, bicyclist or pedestrian. We suggest that Näyhä's contradicting result on fatalities is due to different sampling, non-optimal setting and chance in a fairly small data. However, this does not imply a non-existent effect on accident risk as no exposure-to-risk data [18] are available. People who are anxious of "Black Friday" may stay home, or at least avoid driving a car. The only relevant data [4], suggesting a small decrease in highway traffic, is rather limited and should be confirmed with more extensive research.

## Competing interests

The authors declare that they have no competing interests.

## Authors' contributions

Both authors participated in each stage of research and manuscript preparation.

## Pre-publication history

The pre-publication history for this paper can be accessed here:


